# De Novo Onset of Myasthenia Gravis after Kidney Transplantation

**DOI:** 10.1155/2024/5473862

**Published:** 2024-01-29

**Authors:** Aaron de Souza, Rajesh Raj

**Affiliations:** College of Health & Medicine, University of Tasmania & Launceston General Hospital, Launceston, Australia

## Abstract

Myasthenia gravis occurring de novo after kidney transplantation is a rare course of severe muscle weakness. A 57-year-old female on treatment with peritoneal dialysis following polycystic kidney disease received a renal transplant with standard basiliximab induction. She had no preceding history of neuromuscular problems. Three months after transplant she presented with progressive weakness and fatigability, finally needing a wheelchair to mobilise. Graft function was stable. Examination revealed patchy limb weakness, worsening on repeated exercise. There were no abnormalities in cranial nerves, reflexes, or sensation. Electromyography was normal, but repetitive nerve stimulation studies showed a postsynaptic neuromuscular transmission defect suggestive of myasthenia gravis. Serological testing revealed no putative antibodies. Initial treatment with pyridostigmine was not tolerated. Following an episode of hospitalisation with severe limb weakness, she received intravenous immunoglobulin and showed dramatic improvement, which persisted over the next few weeks. Approximately 6 months later, she had a relapse of her symptoms, which once again responded to intravenous immunoglobulin therapy. De novo myasthenia gravis after transplantation is a rare entity, infrequently reported in the literature. This illness is surprising since immunosuppression after transplant is usually sufficient to prevent immune-mediated disease. This patient had no history of similar illnesses. Delayed physical recovery after major surgery such as renal transplantation is often attributed to other causes such as deconditioning, and patients are often prescribed physiotherapy as a response. In this patient, the profound unexplained weakness that persisted for several weeks after transplant prompted referral to the neurologist, which enabled this rare diagnosis to be made. This story highlights the need to monitor unexpected symptoms closely and to consider a wide differential diagnosis when improvement after transplant is not along usual expected lines. Finally, this case also illustrates the benefits of multidisciplinary involvement in the care of these complex patients.

## 1. Introduction

Transplantation surgery often successfully restores kidney function, but the full physical recovery of the transplant recipient may lag behind. This disparity may be more pronounced in older patients and in those with multiple other illnesses, where the pretransplant functional status and lifestyle may modify the trajectory of recovery [1]. Complications after surgery and the effects of immunosuppression may also contribute (see Table 1). However, most patients are expected to make a gradual recovery in the weeks after transplantation. Especially when supported with appropriate physiotherapy programmes, transplant recipients can expect to have significant functional improvement in the year after transplant [2, 3].

Occasionally, however, physical recovery does not match expectations despite patients regaining adequate kidney function. After common causes as listed in Table 1 are addressed, alternate diagnoses may need to be considered. This report presents a patient with a new onset of fatigable limb-girdle weakness due to seronegative myasthenia gravis occurring early after a kidney transplant, who responded satisfactorily to further immunomodulation with intravenous immunoglobulin. Written informed consent was obtained from the patient prior to the publication of this case report.

## 2. Case Presentation

A 57-year-old woman with polycystic kidney disease and end-stage kidney disease underwent a cadaveric renal transplant after 3 years of peritoneal dialysis treatment. Prior to surgery, she had an excellent functional status, with no history of any neuromuscular problems. She received a 5/6 HLA mismatched, ABO-compatible transplant with basiliximab induction, and subsequent immunosuppression with tacrolimus (initial target levels achieved 8–9 *μ*g/L), mycophenolate mofetil, and prednisolone. Kidney function gradually improved, and her creatinine reached a baseline value of 160 *μ*mol/L by week 10.

Three weeks after surgery, she noted gradual onset of lower limb weakness with shortness of breath on exertion. She later developed upper limb weakness as well. She reported diurnal fluctuations, feeling better in the morning, and being more tired or as the day wore on. She denied difficulties with vision, swallowing, or speaking. Sensory, bowel, and bladder functions were normal. No electrolyte abnormalities were detected; renal function remained stable. Her symptoms worsened, and by the third month after transplant, she required assistance with most activities at home and needed a wheelchair for mobility. She was referred to the neurologist.

At the neurology review, now twelve weeks after transplant, proximal upper and lower limb weakness in a nonselective pattern was noted, worsening on repeated exercise for 15 seconds. She had trouble getting up from a chair and stood with knees hyperextended. Her gait had a waddling character, and she became short of breath after walking for four minutes. Cranial nerve function was normal without fatigable ptosis or diplopia and a strong cough. Reflexes, sensation, and coordination were normal.

Serum creatine kinase, myositis antibody panel, anti-hydroxymethylglutarate-coenzyme A reductase antibody, serum thyroid-stimulating hormone, and vitamin D levels were all normal. Electromyography (EMG) demonstrated normal peripheral motor and sensory nerve function with no abnormal spontaneous activity on needle EMG. Repetitive nerve stimulation (RNS) studies were consistent with a postsynaptic neuromuscular transmission defect, with a decrement of 10.4-15% on 2 Hz stimulation of the spinal accessory and facial nerves, with postexercise exhaustion. There was no postexercise increment. Needle electromyography (EMG) showed occasional unstable low-amplitude short-duration polyphasic motor unit action potentials with complete recruitment, consistent with a neuromuscular junction disorder and excluding a neurogenic process, myotonia, or myopathy. [4]. A provisional diagnosis of myasthenia gravis was made. Antiacetylcholine receptor (AChR) and anti-muscle-specific tyrosine kinase (MuSK) antibodies were absent in serum.

The patient did not tolerate initial treatment with pyridostigmine and ceased it. She was admitted to hospital soon thereafter with profuse diarrhoea, becoming bedbound due to worsening of limb weakness. She received intravenous immunoglobulin at 2 g/kg over five days and noted significant improvement in muscle power and fatigability. She became ambulant without aid over the next month. The patient was delighted with the outcome and stated that it was only after she regained her physical independence did she feel that transplantation was the right choice for her. Six months later, she had a relapse of her limb weakness, and a decrement of 8.5 to 19.8% was again noted on RNS. Further immunoglobulin therapy at 1 g/kg and maintenance at 0.6 g/kg every 4 weeks thereafter again produced dramatic relief.

## 3. Discussion

Myasthenia gravis is a relatively uncommon cause of ocular, bulbar, and limb girdle weakness and, in adults, is often due to an acquired autoimmune postsynaptic disorder of the neuromuscular junction. While exacerbations of existing myasthenic weakness due to intercurrent medical illness, surgery, certain medications, or intensive care are well known, there have been only two reports of myasthenia developing de novo after solid organ transplantation [5, 6]. Myasthenia gravis is associated with antibodies to the nicotinic AChR in 80% and to MuSK in 5%, although around 15% remain double-seronegative [7]. The failure to detect these biomarkers in our patient may be considered a limitation of the case report. However, the diagnosis of an immune-mediated postsynaptic neuromuscular junction transmission disorder is reasonable, given positive results from two RNS studies and the response to immunomodulation. Unusually, our patient developed myasthenia while on immunosuppression, which is itself often employed to treat this immune-mediated condition.

The aetiopathogenesis of myasthenia in our patient is unclear, although her altered immune status during the period of intense immunosuppression after transplantation, the surgical procedure itself, and exposure to multiple medications during the perioperative period are all likely to be relevant [8]. Reports of de novo myasthenia gravis after solid organ transplants are rare—a similar patient after kidney transplantation reported by Nieto-Ríos et al. had received alemtuzumab for induction of immunosuppression unlike our patient, who received basiliximab [5]. Alemtuzumab therapy leads to a surge of immature B-cell subsets in the absence of T-cell-mediated regulation, which may give rise to autoreactive B-cells and subsequently to antibody-driven autoimmune disease months or years after exposure [9]. This has not been reported with basiliximab, which is in fact used for resistant myasthenia gravis [10]. Similarly, reports of myasthenia gravis following bone marrow transplantation may be related to the progressive development of donor-derived immunity, either through chronic graft-versus-host disease producing autoreactive T-cells and autoantibodies, or via adoptive immunity mediated by the introduction of sensitised lymphocytes from the donor [11].

Neurological complications appearing after transplant are often side effects related to medications, including immunosuppressants. Tremors, toxic peripheral neuropathies, seizures, psychiatric disorders, and myopathy are well described and attributed to various drugs [8]. De novo myasthenic syndromes are extremely rare, and their pathogenesis in this setting remains uncertain.

## 4. Conclusion

Weakness and fatigability are common after major surgery such as organ transplantation, but the diagnosis of a new-onset neuromuscular junction disorder, as in our patient, is unexpected. While all patients are monitored closely after transplantation, primary attention is often directed to the transplanted organ itself. This case report highlights the importance of considering multiple differentials when unusual presentations arise and demonstrates the value of multidisciplinary involvement in complex patients.

## Figures and Tables

**Table 1:** Causes of delayed physical recovery in the early post-transplantation period.

Pretransplant status
Reduced muscle mass/frailty
Bone-mineral disorders and complications
Chronic fatigue
Anaemia
Nutritional deficiencies
Obesity
Lifestyle factors
Physical deconditioning from multiple comorbidities
Pre-existing neurological illness
Other contributors
Surgical/postoperative complications
Delayed graft function/need for ongoing dialysis
Complications of immunosuppression including infections
Effects of other medications such as statins or opioids
Metabolic causes/dyselectrolytemias
Psychological factors

## Data Availability

Patient data included in this case report can be made available in a deidentified format by contacting the authors.
